# From a Medicinal Mushroom Blend a Direct Anticancer Effect on Triple-Negative Breast Cancer: A Preclinical Study on Lung Metastases

**DOI:** 10.3390/molecules25225400

**Published:** 2020-11-18

**Authors:** Elisa Roda, Fabrizio De Luca, Carlo Alessandro Locatelli, Daniela Ratto, Carmine Di Iorio, Elena Savino, Maria Grazia Bottone, Paola Rossi

**Affiliations:** 1 Department of Biology and Biotechnology “L. Spallanzani”, University of Pavia, 27100 Pavia, Italy; fabrizio.deluca01@universitadipavia.it (F.D.L.); daniela.ratto01@universitadipavia.it (D.R.); carmine.diiorio01@universitadipavia.it (C.D.I.); mariagrazia.bottone@unipv.it (M.G.B.); 2 Laboratory of Clinical & Experimental Toxicology, Pavia Poison Centre, National Toxicology Information Centre, Toxicology Unit, Istituti Clinici Scientifici Maugeri IRCCS, 27100 Pavia, Italy; carlo.locatelli@icsmaugeri.it; 3 Department of Earth and Environmental Science, University of Pavia, 27100 Pavia, Italy; elena.savino@unipv.it

**Keywords:** breast cancer, lung metastases, in vivo, apoptosis, complementary medicine, medicinal mushrooms

## Abstract

Bioactive metabolites isolated from medicinal mushrooms (MM) used as supportive treatment in conventional oncology have recently gained interest. Acting as anticancer agents, they interfere with tumor cells and microenvironment (TME), disturbing cancer development/progression. Nonetheless, their action mechanisms still need to be elucidated. Recently, using a 4T1 triple-negative mouse BC model, we demonstrated that supplementation with Micotherapy U-Care, a MM blend, produced a striking reduction of lung metastases density/number, paralleled by decreased inflammation and oxidative stress both in TME and metastases, together with QoL amelioration. We hypothesized that these effects could be due to either a direct anticancer effect and/or to a secondary/indirect impact of Micotherapy U-Care on systemic inflammation/immunomodulation. To address this question, we presently focused on apoptosis/proliferation, investigating specific molecules, i.e., PARP1, p53, BAX, Bcl2, and PCNA, whose critical role in BC is well recognized. We revealed that Micotherapy U-Care is effective to influence balance between cell death and proliferation, which appeared strictly interconnected and inversely related (p53/Bax vs. Bcl2/PARP1/PCNA expression trends). MM blend displayed a direct effect, with different efficacy extent on cancer cells and TME, forcing tumor cells to apoptosis. Yet again, this study supports the potential of MM extracts, as adjuvant supplement in the TNBC management.

## 1. Introduction

Breast cancer (BC) is the most frequently diagnosed malignant neoplasm in women [1]. Due to the progress achieved both in early diagnosis and in novel therapeutic treatments, the death rate of BC is progressively decreasing, but BC still remains one of the leading causes of morbidity and mortality in females worldwide [2]. In particular, in Western countries BC metastases are the second cause of mortality among tumor patients [3,4].

Triple-negative breast cancer (TNBC) is characterized by the lack of expression of specific receptors, i.e., estrogens, progesterone, and epidermal growth factor 2, and represents about 15–20% of all BC diagnoses. Indeed, TNBC differs from other subgroups of BC for its increased growth and fast spreading, with reduced treatment possibilities (due to the absence of the three above reported receptors) and a worse outcome [5,6]. Actually, TNBC patients are extremely prone to metastasis and relapse [7] which mainly affect brain, liver, and, in particular, lungs [8]. Tumor progression to metastasis is a complex and many-sided process, affected by both intrinsic cellular mutational burden and several interactions between malignant and non-malignant cell types, and also constantly regulated by the various extrinsic microenvironmental niches [9].

The tumor microenvironment (TME), consisting of immune cells, fibroblasts, satellite cells, as well as blood and lymphatic vessels, plays a fundamental role in the tumor biology, i.e., the behavior of a bulk tumor, co-evolving in a delicate ecosystem with the tumor niche. This never-ending evolving process is governed by local and distant microenvironments, being also regulated by systemic inflammation. In fact, under cytokines, growth factors, and chemotactic stimuli induction, cancer cells recruit and transform stromal fibroblasts into malignant cells, which alter the extracellular matrix by secreting tumor-stimulating factors to facilitate tumor invasion and metastasis. Thus, based on the strict interaction between tumor cells and multicellular proinflammatory TME, cancer typical features also include the evasion of immune destruction, tumor promoting inflammation, and angiogenesis induction. Indeed, nowadays it is well-known that TME plays a crucial role in cancer progression, therapeutic response, and patient outcome [9,10,11]. Immunotherapy is a recent approach in cancer therapy, specifically addressed on TME [12]. Currently, the elite therapy for treating TNBC is the cytotoxic chemotherapy, characterized by several side effects [13,14]. A bulk of literature demonstrated that chemotherapy induces apoptosis and cancer is a pathology characterized by a dysregulation in cell cycle/death, in which the disruption of the apoptotic pathway leads to uncontrolled cell proliferation [15]. Anomalies in the apoptotic pathway are crucial for cancer genesis, evolution, and regression after treatment [16]. Indeed, the apoptosis rate is crucial in determining the fate between cancer progression and regression as well as in response to current available treatments, i.e., chemotherapy, radiotherapy, surgery, and hormonal therapies.

As a matter of fact, cells undergo apoptosis by changing the balance of proapoptotic and antiapoptotic genes [17]. The main actors involved in apoptosis pathway can be reduced to a few crucial proteins largely preserved through species [18]. In humans and mice, these key proteins belong to the Bcl-2 family [18], Poly (ADP-ribose) polymerase (PARP) [19], and p53 [20]. Bcl-2 family contains both inhibitors and promoters of apoptosis [18]. Acting as proapoptotic protein, Bax is an important tumor suppressor factor, and its reduced levels provide tumor cells with a selective survival advantage, contributing to their expansion [21]. Contrarily, acting as antiapoptotic protein, Bcl-2 plays an essential role as a cell survivor enhancer, and its increased expression level gives cancer cells with a selective survival gain [22]. Concerning PARPs, PARP1 is a nuclear protein which contributes in DNA single-strand break repair, and its dysregulation is involved in tumorigenesis phenomena to such a degree that PARP1 inhibitors have been authorized for the treatment of some types of BC, stimulating both apoptosis induction and synthetic lethality mechanism/DNA repair [23,24,25,26,27]. p53 protein is a nuclear protein known as tumor suppression factor, which plays a critical role deciding whether DNA would be repaired or the damaged cell will self-destruct, inducing programmed cell death [20,28]. Dysregulation of these mentioned proteins is a frequent feature of human malignant diseases and causal for therapy resistance. Tumor cells usually escape from apoptosis by downregulation of proapoptotic genes and/or hyperactivation of antiapoptotic genes. Therefore, apoptosis induction in cancer cells is considered an excellent approach for treating tumors [29].

It has also to be mentioned the PCNA pivotal role in cancer, owing to its function in cell proliferation. Since cancer is caused by and manifest through multiple mechanisms, many of which converging to deregulated proliferation at primary and metastatic sites, and PCNA is an indispensable factor for cell cycle control, DNA replication, DNA nucleotide excision repair, and chromatin assembly, PCNA inhibition is considered to be another viable anticancer strategy [30,31].

In the last decades, considerable attention has been focused on developing/identifying new compounds for TNBC treatment with absent/minimal side adverse effects. Interestingly, in recent years the importance of naturally derived compounds has been highlighted as a source of anticancer and proapoptotic drugs [32]. One of the most promising sources for drug discovery in integrative oncology are medicinal mushrooms (MM), which have an established story of use in traditional oriental medicine and as nutritionally functional foods. MM display antitumor, proapoptotic, onco-immunological, and immunomodulatory effects in vitro and improve the quality of life in cancer patients during conventional anticancer treatments [33,34]. Indeed, in the last years, the use of several MM has been approved as adjuvant supplements in antitumor therapy in different countries.

Different MM produce hundreds of bioactive compounds which are able to influence, often in a synergistic way, numerous cancer-related pathways, also modulating cellular targets typically involved in cell proliferation, survival, and angiogenesis [33,35].

Several studies showed that the use of MM extracts or their compounds, alone or combined with conventional anticancer treatments, is safe and beneficial [33,35].

The present investigation is strictly linked to a previous study employing a 4T1 triple-negative mouse BC model to explore the effects of an oral supplementation with “Micotherapy U-care” (M. U-care), a medicinal mushroom blend, consisting of a mixture of 20% extracts of mycelia and sporophores of five MM, i.e., *Agaricus blazei*, *Ophiocordyceps sinensis*, *Ganoderma lucidum*, *Grifola frondosa*, and *Lentinula edodes* [36]. Each of these MM have been shown displays anticancer and immunomodulatory effects, both in vitro and in vivo, and in preclinical and clinical studies [34,37].

Using a syngeneic tumor-bearing mouse model of TNBC, we demonstrated that M. U-Care supplementation, starting before 4T1 cells injection and lasting throughout the whole experimental time (about 3 months), elicited (i) an increase in the quality of life, (ii) a dramatic decrease of lung metastases density and nodules number, and (iii) a substantial decrease of inflammatory and oxidative stress pathways, characterized by a similar protein expression trend in both lung TME and metastases [36]. Based on these results, we hypothesized that the supplement effects could have been ascribable either to a direct M. U-care anticancer effect on lung cells and/or to secondary/indirect impacts of the MM blend on systemic inflammation and immunomodulation. To punctually address this question, in the present study, we focused on the programmed cell death pathway, namely, apoptosis, by investigating specific molecules whose critical role in human BC is well known. With this aim, using immunohistochemistry, we evaluated the expression, localization and changes of the following proteins, i.e., PARP1, p53, Bax, and Bcl2, performing a comparative assessment on lung metastases and pulmonary parenchyma, namely, TME. For the sake of clarity, we focused on the pathological outcomes in the murine pulmonary tissue, as the lung is the one of the distant organs recurrently implicated in typical metastatic pattern of primary TNBC [7]. The apoptotic cell death was also examined by in situ detection of DNA fragmentation using the terminal deoxynucleotidyl-transferase (TUNEL) assay. In addition, based on the notion that proliferative activity of cancer cells is also a crucial prognostic marker in the tumor diagnosis, we investigated the role of Proliferating Cell Nuclear Antigen (PCNA), being a pivotal protein directly related to the degrees of tumor malignancy and diagnosis [38,39].

## 2. Results

**Figure 1:**
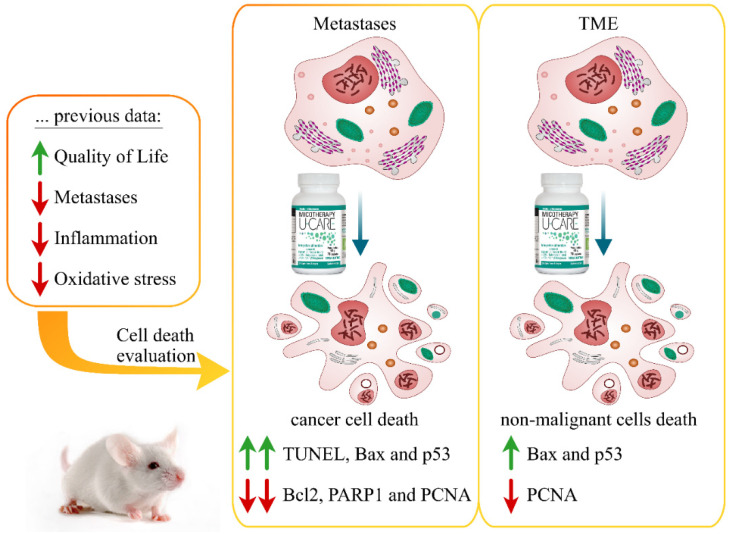
Graphic illustration underlining the main results.

Figure 1 emphasizes the key outcomes of the present investigation and also summarizes some major results of our previous study (for details see the work in [36]).

### 2.1. Raw Materials, Extract Procedure, and Main Active Metabolites of Micotherapy U-Care Blend

The MM blend Micotherapy U-care was produced and supplied by A.V.D. Reform s.r.l. (Noceto, Parma, Italy) and it consists of a mixture of five fungal species, as reported in Table 1.

The specific MM species strains were established by genetic analyses, sequencing ITS regions and confirming the ID code. Each MM was ground, and total genomic DNA was extracted through the DNeasy mini plant kit (Qiagen NV, Venlo, Netherlands). The Internal Transcribed Spacer (ITS) regions of nuclear DNA were amplified by using PCR, applying two set of primers: ITS F 5′-AGAAAGTCGTAACAAGGTTTCCGTAG-3′, ITS R 5′-TTTTCCTCCGCTCATTGATATGCTT-3′, ITS-g F 5′-TCCGTAGGTGAACCTGCGG-3′, and ITS-g R 5′-TCCTCCGCTTATTGATATGC-3′. Next, the PCR products were purified, sequenced by Eurofins Genomics (Konstanz, Germany), and identified by using NCBI Nucleotide Blast software, version 2.9.0 (Table 1, ID code).

Next, the sporophores and/or mycelia were cultivated, harvested, and the fresh material was extracted for 3 h at 95 °C in distilled water with ethanol 10% (for 1 kg of raw material, 15 L of water-ethanol solution was used). After water-ethanol extraction, solid and liquid components were divided, and the fluid part was dehydrated until the humidity amount was smaller than 7%. Dry extracts were ground and blended to have the 20% of each selected mushroom in the MM blend Micotheraphy U-care (Table 1).

Finally, the polysaccharide content of Micotherapy U-care was determined by using a β-Glucan Assay Kit, and the polysaccharide content was more than 30%. Of this 30%, more than 15% of the polysaccharides were 1,3-1,6 β-glucans, the main active metabolites in Micotherapy U-care.

### 2.2. TUNEL Assay

**Figure 2:**
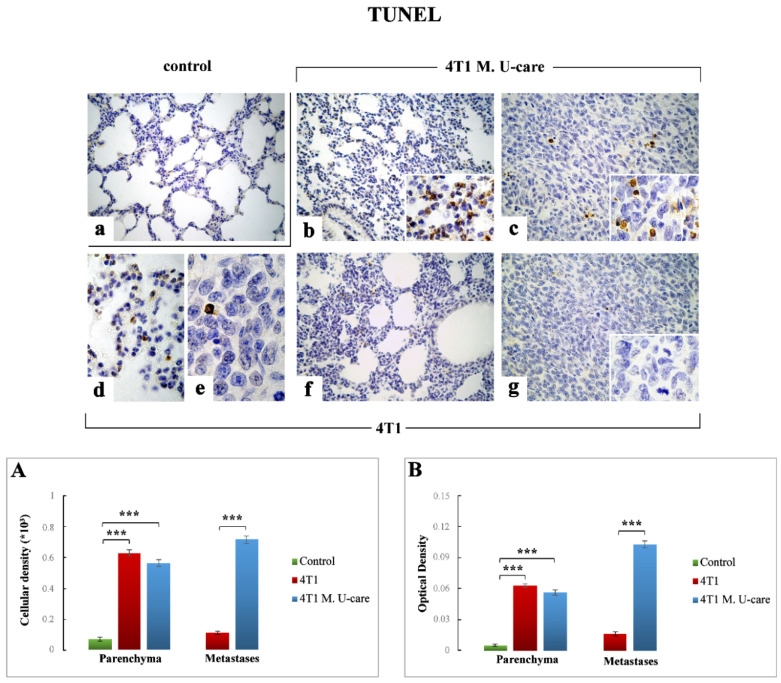
Terminal deoxynucleotidyl-transferase (TUNEL) immunostaining in healthy control (**a**), 4T1 M. U-care (**b**,**c**) and 4T1 (**d**–**g**) mice. Light microscopy magnification: 40× (**a**–**c**,**f**,**g**); 100× (**d**,**e**, insert in panels (**b**,**c**,**g**)). Panel (**A**) and (**B**): Histograms presenting immunopositive cell density and OD, respectively. *p* values calculated by unpaired Student’s *t*-test: (***) < 0.001.

TUNEL staining, as a typical marker of apoptotic events [40], revealed an extensive spreading in alveolar pneumocytes (both type I and II) and bronchiolar epithelial cells as well as in metastatic nodules (Figure 2).

In particular, concerning the TME, TUNEL-immunoreactivity significantly increased in both 4T1 and 4T1 M. U-care mice (Figure 2b,d,f, respectively) compared to controls (Figure 2a).

The quantitative analysis showed an extremely significant increase of TUNEL-immunopositive cell density and OD comparing 4T1 animals to controls (0.63 ± 0.02 vs. 0.07 ± 0.01 and 0.06 ± 0.00 vs. 0.01 ± 0.00, for cell density and OD, respectively) (Figure 2, Panels (A,B)). In a similar manner, an extremely significant immunoreactivity enhancement was detected when comparing 4T1 M. U-care mice to controls (0.56 ± 0.02 vs. 0.07 ± 0.01 and 0.06 ± 0.00 vs. 0.01 ± 0.00, for cell density and OD, respectively). Diversely, a slight decrease of TUNEL-immunopositive cell density and OD was revealed in 4T1 M. U-care animals compared to 4T1 mice (0.56 ± 0.02 vs. 0.63 ± 0.02 and 0.06 ± 0.00 vs. 0.06 ± 0.00, for cell density and OD, respectively) (Figure 2, Panels (A,B)).

Regarding metastases, an extremely significant increase of TUNEL-immunopositive cell density and OD was detected in 4T1 M. U-care mice compared to 4T1 animals (Figure 2c,e,g, respectively): 0.72 ± 0.02 vs. 0.11 ± 0.01 and 0.10 ± 0.00 vs. 0.02 ± 0.00, for density and OD, respectively (Figure 2, Panels (A,B)). Notably, in 4T1 mice several mitoses were also observable (Figure 2, insert in Figure 2g).

### 2.3. PARP1, p53, Bax, Bcl2, and PCNA Immunohistochemical Assessment

The cellular expression, localization, and distribution of PARP1, p53, Bax, Bcl2, and PCNA, all involved in cell death and proliferation pathways, were explored.

The immunohistochemical evaluation of all these molecules revealed a widespread labeling in the metastatic nodules and/or in TME, at bronchiolar and alveolar level, evidencing a different efficacy of the MM blend, with the more marked effect on tumor cells.

#### 2.3.1. PARP1

**Figure 3:**
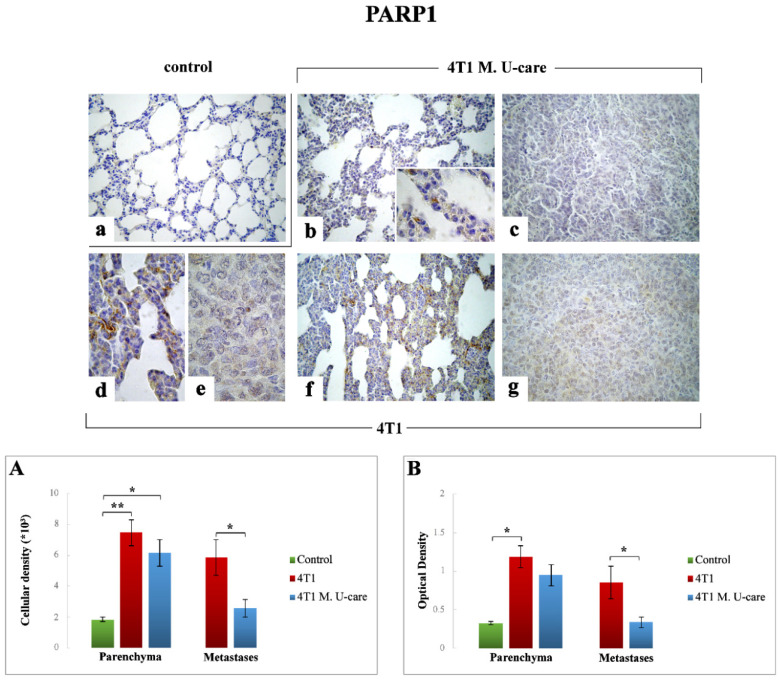
Immunostaining reaction for PARP1 in healthy control (**a**), 4T1 M. U-care (**b**,**c**) and 4T1 (**d**–**g**) mice. Light microscopy magnification: 40× (**a**–**c**,**f**,**g**); 100× (**d**,**e**, insert in panel (**b**)). Panels (**A**) and (**B**): Histograms displaying immunopositive cell density and OD, respectively. *p* values calculated by Unpaired Student’s *t*-test: (*) < 0.05 and (**) < 0.01.

A very significant increase of PARP1-immunoreactive cell density was measured in the TME of 4T1 animals (Figure 3d,f) compared to controls (Figure 2a): 7.46 ± 0.83 vs. 1.85 ± 0.14, respectively (Figure 3, Panel (A)); similarly, a significant increase was detected when comparing 4T1 M. U-care mice (Figure 3b) to controls (6.14 ± 0.87 vs. 1.85 ± 0.14, respectively). Notably, any difference was determined evaluating PARP1-immunopositive cell density in 4T1 animals and 4T1 M. U-care mice (7.46 ± 0.83 vs. 6.14 ± 0.87, respectively) (Figure 3, Panel (A)).

Likewise, a significant increase of PARP1-immunoreactive OD was measured in 4T1 animals compared to controls (1.19 ± 0.14 vs. 0.32 ± 0.02, respectively) (Figure 3, Panel (B)), while, differently, any difference was revealed evaluating PARP1-immunopositive OD in 4T1 animals compared to 4T1 M. U-care mice (1.19 ± 0.14 vs. 0.95 ± 0.14, respectively), or even between 4T1 M. U-care and controls (0.95 ± 0.14 vs. 0.32 ± 0.02, respectively) (Figure 3, Panel (B)).

Concerning the metastatic tissue, a significant increase of PARP1-immunopositive cell density and OD was determined in 4T1 mice compared to 4T1 M. U-care animals (5.85 ± 1.16 vs. 2.57 ± 0.57 and 0.85 ± 0.21 vs. 0.34 ± 0.07, for cell density and OD, respectively) (Figure 3, Panels (A,B)).

#### 2.3.2. p53

**Figure 4:**
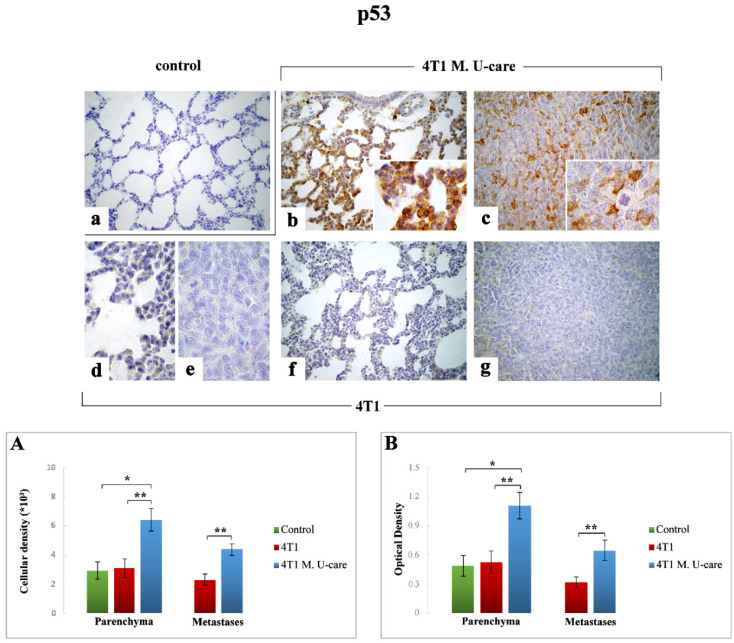
p53-immunostaining reaction in healthy control (**a**), 4T1 M. U-care (**b**,**c**) and 4T1 (**d**–**g**) mice. Light microscopy magnification: 40× (**a**–**c**,**f**,**g**); 100× (**d**,**e**, insert in panels (**b**,**c**)). Panels (**A**) and (**B**): Histograms showing immunopositive cell density and OD, respectively. *p* values calculated by Unpaired Student’s *t*-test: (*) < 0.05 and (**) < 0.01.

Comparably to the above reported TUNEL immunostaining trend, p53 immunoreactivity was significantly increased in 4T1 M. U-care mice (Figure 4b,c) compared to both 4T1 animals (Figure 4d–g) and controls (Figure 4a). Specifically, a very significant increase of p53-immunopositive cell density and OD was determined in 4T1 M. U-care animals compared to 4T1 mice (6.42 ± 0.78 vs. 3.09 ± 0.65 and 1.11 ± 0.14 vs. 0.53 ± 0.12, for cell density and OD, respectively). A significant increase of p53-immunopositive cell density and OD was also observed when comparing 4T1 M. U-care animals to control (6.42 ± 0.78 vs. 2.93 ± 0.59 and 1.11 ± 0.14 vs. 0.49 ± 0.11, for cell density and OD, respectively). Differently, any significant difference was calculated when comparing 4T1 mice to controls (3.09 ± 0.65 vs. 2.93 ± 0.59 and 0.53 ± 0.12 vs. 0.49 ± 0.11, for cell density and OD, respectively) (Figure 4, Panel (A,B)).

With regard to metastatic tissue, a very significant increase of both p53-immunoreactive cell density and OD was determined in 4T1 M. U-care mice compared to 4T1 animals (4.38 ± 0.63 vs. 2.29 ± 0.39 and 0.64 ± 0.10 vs. 0.32 ± 0.06, for density and OD, respectively) (Figure 4, Panels (A,B)).

#### 2.3.3. Bax

**Figure 5:**
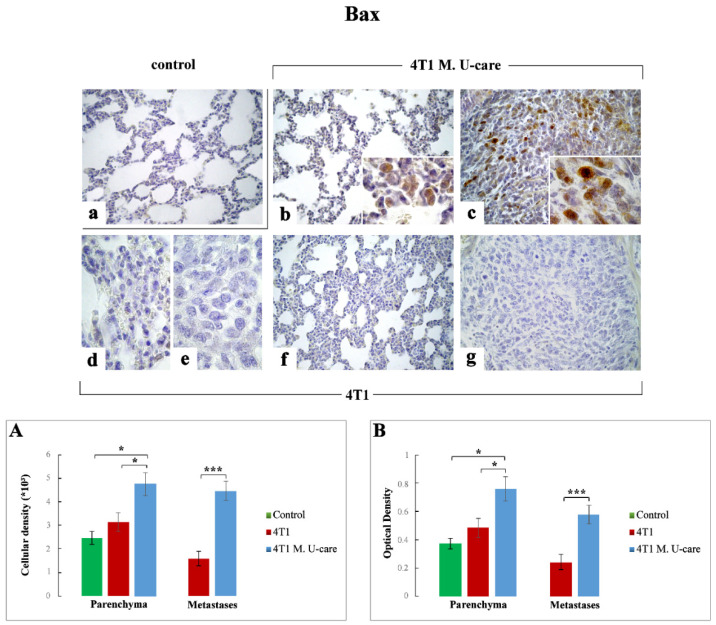
Immunohistochemical staining for Bax in healthy control (**a**), 4T1 M. U-care (**b**,**c**) and 4T1 (**d**–**g**) mice. Light microscopy magnification: 40× (**a**–**c**,**f**,**g**); 100× (**d**,**e**, insert in panels (**b**,**c**)). Panels (**A**) and (**B**): Histograms showing immunopositive cell density and OD, respectively. *p* values calculated by Unpaired Student’s *t*-test: (*) < 0.05 and (***) < 0.001.

Similarly to p53, a significant increase in Bax immunopositivity was observed in the TME of 4T1 M. U-care mice at alveolar and stromal level, with several immunopositive endothelial cells in bronchiolar areas (Figure 5b), compared to both 4T1 mice (Figure 5d,f) and controls (Figure 5a). In the same manner, Bax resulted overexpressed in metastatic nodules 4T1 M. U-care mice (Figure 5c), compared to 4T1 animals (Figure 5e,g). Notably, a significant increase of Bax-immunoreactive cell density was measured in 4T1 M. U-care animals compared to 4T1 mice (4.75 ± 0.48 vs. 3.13 ± 0.40, respectively). Likewise, a significant increase was observed in 4T1 M. U-care mice compared to control (4.75 ± 0.48 vs. 2.47 ± 0.28, respectively), while any difference was determined when comparing 4T1 animals and controls (3.13 ± 0.4 and 2.47 ± 0.28, respectively) (Figure 5, Panel (A)). Moreover, a significant increase of Bax-immunostaining OD was evidenced in 4T1 M. U-care mice, compared to both 4T1 and controls (0.76 ± 0.09 vs. 0.48 ± 0.07 and 0.76 ± 0.09 vs. 0.37 ± 0.04, respectively). Any significant difference was measured when comparing 4T1 mice to controls (0.48 ± 0.07 vs. 0.37 ± 0.04, respectively) (Figure 5, Panel (B)).

Concerning the metastases, an extremely significant increase of both Bax-immunopositive cell density and OD was determined in 4T1 M. U-care mice compared to 4T1 animals (4.47 ± 0.40 vs. 1.59 ± 0.31, and 0.58 ± 0.07 vs. 0.24 ± 0.06, respectively) (Figure 5, Panels (A,B)).

#### 2.3.4. Bcl2

**Figure 6:**
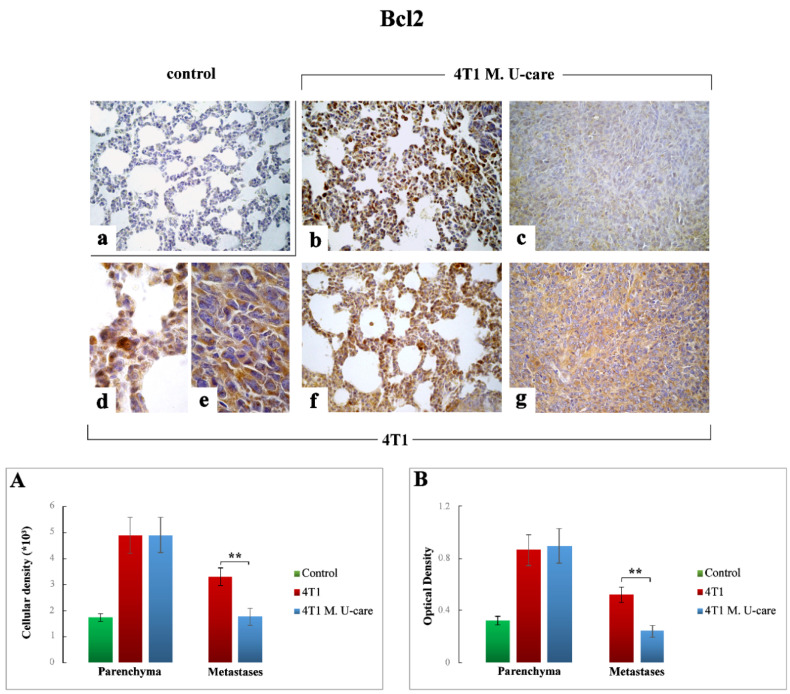
Bcl2-immunostaining reaction in healthy control (**a**), 4T1 M. U-care (**b**,**c**) and 4T1 (**d**–**g**) mice. Light microscopy magnification: 40× (**a**–**c**,**f**,**g**); 100× (**d**,**e**). Panels (**A**) and (**B**): Histograms showing immunopositive cell density and OD, respectively. *p* values calculated by Unpaired Student’s *t*-test: (**) < 0.01.

Concerning the TME, Bcl2-immunoreactivity appeared slightly enhanced in both 4T1 and 4T1 M. U-care mice (Figure 6b,d,f, respectively) compared to controls (Figure 6a). Notably, a slight non-significant increase of Bcl2-immunoreactive cell density and OD was measured both in 4T1 and 4T1 M. U-care animals compared to controls (4.88 ± 0.69 vs. 4.89 ± 0.68 vs. 1.74 ± 0.15 and 0.86 ± 0.12 vs. 0.89 ± 0.13 vs. 0.32 ± 0.03, for density and OD, respectively). Any difference was evidenced evaluating Bcl2-immunopositive cell density and OD in 4T1 animals and 4T1 M. U-care mice (4.88 ± 0.69 vs. 4.89 ± 0.68 and 0.86 ± 0.12 vs. 0.89 ± 0.13, for density and OD, respectively) (Figure 6, Panels (A,B)).

Regarding the metastatic nodules (Figure 6c,e,g), contrarily to Bax, a very significant decrease of Bcl2-immunopositive cell density and OD was detected in 4T1 M. U-care mice compared to 4T1 (1.77 ± 0.32 vs. 3.29 ± 0.34 and 0.24 ± 0.04 vs. 0.52 ± 0.06, for density and OD, respectively) (Figure 6, Panels (A,B)).

#### 2.3.5. PCNA

**Figure 7:**
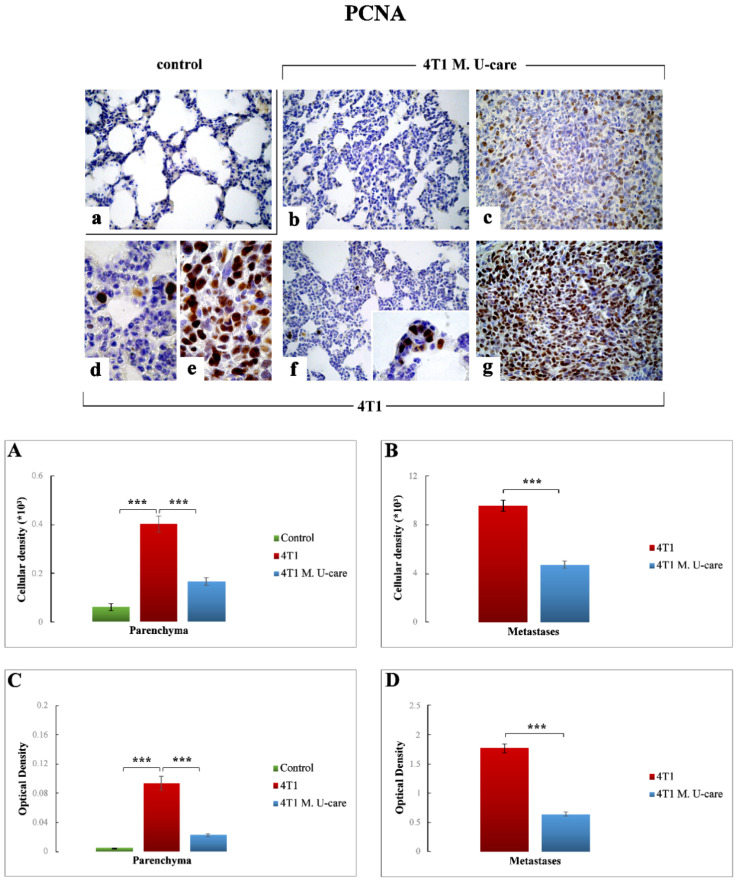
DAB-immunostaining reaction for PCNA in healthy control (**a**) 4T1 M. U-care (**b**,**c**), and 4T1 (**d**–**g**) mice. Light microscopy magnification: 40× (**a**–**c**,**f**,**g**); 100× (**d**,**e**, insert in panel (**f**)). Panels (**A**) and (**B**): Histograms showing immunopositive cell density. Panels (**C**) and (**D**): histograms exhibiting immunoreactive OD. *p* values calculated by Unpaired Student’s *t*-test: (***) < 0.001.

PCNA-immunopositivity was extremely enhanced in TME of 4T1 mice (Figure 7d,f) compared to both 4T1 M. U-care (Figure 7b) and controls (Figure 7a). In particular, the quantitative analysis highlighted an extremely significant increase in PCNA-immunoreactive cell density and OD when comparing 4T1 animals to controls (0.40 ± 0.03 vs. 0.06 ± 0.01 and 0.93 ± 0.01 vs. 0.01 ± 0.00, for cell density and OD, respectively). In a similar manner, an extremely significant enhancement was detected when comparing 4T1 mice to 4T1 M. U-care animals (0.40 ± 0.03 vs. 0.17 ± 0.02 and 0.93 ± 0.01 vs. 0.02 ± 0.00, for cell density and OD, respectively). No significant differences were revealed in 4T1 M. U-care animals compared to control (0.17 ± 0.02 vs. 0.06 ± 0.01 and 0.02 ± 0.00 vs. 0.01 ± 0.00, for cell density and OD, respectively) (Figure 7, Panels (A,B)).

Concerning the metastatic tissue, an extremely significant decrease of PCNA-immunopositive cell density and OD was detected in 4T1 M. U-care mice (Figure 7c) compared to 4T1 animals (Figure 7e,g): 4.74 ± 0.28 vs. 9.57 ± 0.44 and 0.64 ± 0.04 vs. 1.77 ± 0.08, for density and OD, respectively (Figure 7, Panels (A,B)).

## 3. Discussion

Tumor development and progression are influenced by rearrangement of TME components, e.g., immune cells, fibroblasts, satellite cells, blood, and lymphatic vessels. Tumor cells are known to manipulate the function of cellular and non-cellular components through a complex signaling network to gain tumorigenesis, tumor maintenance, and drug resistance (MDR), taking advantage of the non-malignant cells. Experimental and clinical data evidence that an in-depth analysis of the bidirectional communications and interactions between tumor cells and their surrounding dynamic TME is essential to identify the existing mechanisms of tumor expansion and invasion [41]. This complex network moreover involved apoptosis, also engaged to efficiently eliminate dysfunctional cells, plays an important role in both carcinogenesis and cancer treatment [42]. In particular, to guarantee its nourished growth, cancer is able to provide both endurance signals as well as mechanisms saving malignant cells from apoptosis. It is well known that an imbalance between cell proliferation and cell death typically characterizes cancer condition, in which several genetic aberrations may drive malignant cells to an uncontrolled progression and survival [43,44].

In our previous paper using the same preclinical model, i.e., 4T1 triple-negative mouse BC, we demonstrated that M. U-Care supplementation, starting 2 months before 4T1 injection and lasting throughout the whole experimental time (including tumor development and metastatization), produced a dramatic reduction of both lung metastases density and number. These effects were accompanied by a substantial decrease of inflammation and oxidative stress both in lung TME and metastases, together with a bettering of the QoL [36]. Therefore, we hypothesized that the supplement effects could have been ascribable either to a direct anticancer effect and/or to the secondary/indirect impacts of the MM blend on systemic inflammation and immunomodulation. Therefore, aiming at addressing this crucial question, in the present investigation, we focused on the programmed cell death, investigating specific molecules, i.e., PARP1, p53, BAX, and Bcl2, pivotally involved in apoptotic pathway, whose critical role in human BC is well known. Further, in parallel we investigated PCNA expression pathway, based on the great interest on the pivotal role of PCNA in cancer cells proliferation, also taking into consideration that PCNA modifications may determine both tumor progression as well as the outcome of anticancer treatment.

Firstly, it has to be highlighted that performing a comparative assessment on cancer tissue, i.e., lung metastases, and surrounding TME by TUNEL assay, we revealed the existence of a different expression trend in 4T1 animals compared to 4T1 M. U-care mice. In detail, in these latter animals, an extremely intense immunopositivity was observed in the metastases, indicating an evident activation of the apoptotic pathway in cancer cells after the oral supplementation with the MM blend. Interestingly, this effect was definitely weaker in 4T1 animals, in which several mitoses were clearly observed in the tumor nodules, thus demonstrating the occurrence of an already active proliferation process. Concerning the TME, any statistical difference was measured comparing 4T1 M. U-care animals and 4T1 mice. These data corroborate the action of the MM blend, who display a powerful action able to drive metastatic cells to apoptosis, whereas was less effective with regards to the surrounding TME.

Concerning all evaluated apoptotic and proliferation markers, the comparative assessment of TME and metastases revealed diverse trends of protein expression, with the metastatic nodules being the most affected.

With regards to PARP1 levels, crucially implicated in tumorigenesis phenomena [24,26,27], the significant reduction observed in metastatic nodules of 4T1 M. U-care mice compared to 4T1 animals seemed to demonstrate a valuable action of the adjuvant micotherapic supplementation, suggesting a direct influence of the blend on tumor cells.

Our putative idea of a direct MM blend action able to determine an imbalance between proliferation and apoptosis, driving to a significant increase in apoptotic events, was further supported by p53 and Bax high expression levels measured in 4T1 M. U-care mice, showing the most striking effect in the metastatic nodules. By contrast, concerning both Bax and p53, an almost complete lack of effect was determined in 4T1 animals, in which the protein expression levels were similar to that observed in controls. Acting as proapoptotic protein, Bax should essentially play as a tumor suppressor factor. Consequently, reductions in its expression levels would provide tumor cells with a selective survival advantage, contributing to their expansion and invasion [21]. In particular, the reduced Bax expression levels can be traced back to an alteration of the p53 function which, by itself, is able to alter the levels of this proapoptotic protein [21]. Certainly, TP53 mutations are the most common genetic alterations in breast cancer [28]. Notably, the increased immunopositivity for the wild type p53 isoform detected in 4T1 M. U-care mice could indicate a possible attempt to cell cycle arrest, in order to allow cellular repair processes and inhibit the proliferation of damaged cells.

Based on the notion that (i) several apoptotic stimuli induce cell death through a Bcl-2-regulated pathway and (ii) proteins belonging to Bcl2 family can play a dichotomous role, acting both as promoters as well as inhibitors of apoptotic events [21,45,46], we may suppose that the lower expression of Bcl-2 determined in metastatic tissue of 4T1 M. U-care mice could be related to a decrease in cancer cell proliferation, evidencing an alteration of the proliferation/apoptosis balance. This effect could also counteract the capacity of Bcl2 to trigger drug resistance at high expression [21,47]. Moreover, the overexpression of p53 in 4T1 M. U-Care mice may support a possible role of p53 in downregulating Bcl-2, feasibly explaining the apoptosis induction by wild type p53. Notably, it has to be underlined that, differently to all other evaluated markers, but similarly to TUNEL staining, we determined any difference in the Bcl-2 expression trend comparing metastases and TME in 4T1 M. U-care mice and 4T1 animals. Specifically, TME appeared to be unaffected by the mycotherapic oral supplementation, whereas the MM blend display a specific and selective effect restricted to the metastatic area.

In addition, concerning the proliferation marker PCNA, we evidenced a reduced expression in metastatic nodules in 4T1 M. U-care mice, thus highlighting an evident inhibitory effect of the MM blend on proliferation activity in tumor cells. Notably, a strong positive correlation between the expression of PCNA and COX2 in breast cancer has been recently highlighted [48], also in accordance with our previous findings [36].

Taken together the present data supported that the oral supplementation is effective to induce a peculiar swinging balance of cell death and proliferation, in which these two essential mechanisms appeared strictly interconnected and inversely related (see p53 and Bax vs. Bcl2 and PCNA expression trends). Notably, the MM blend bettered the cancer state with a direct effect, able to force tumor metastatic cells to an apoptotic fate.

In summary, all the present findings confirmed the protective role played by Micotherapy U-care blend in the lung metastases and surrounding TME. Our data further suggest that both an immunomodulatory anti-inflammatory systemic effect and a direct, selective anticancer effect act in a positive pleiotropic way. This double action mechanism, which (i) disturbs the TME signaling and (ii) targets the complex apoptotic pathway, could represent a promising approach for patient’s treatment. In fact, in the search for novel therapeutic agents targeting tumor development and progression, MM-derived natural bioactive compounds, displaying multi-targeting potential, can overcome the disadvantages of monotherapy such as side effects and drug resistance. In particular, TNBC is the most aggressive malignant BC, difficult to treat due to its unresponsiveness to current clinical targeted treatments (e.g., hormonal therapy protocols or chemotherapeutics targeting HER2 protein receptors) and high rate of recurrence. In this scenario, the biological effects of combining M. U-care supplement with conventional therapies to target crucial cancer signaling pathways, i.e., proliferation and apoptosis, may interfere with cellular and molecular processes fueling TNBC growth, trying to create a new joint medical protocol able to hinder the typical TNBC metastatic pattern, i.e., frequent occurrence of distant metastases, mainly localized in lung, central nervous system, and bones, often associated with poor prognosis.

## 4. Materials and Methods

### 4.1. Cell Culture

The mice breast cancer cell line, 4T1, was acquired from American Type Culture Collection (ATCC) and maintained at 37 °C in a humidified atmosphere (95% air/5% CO_2_) [36].

### 4.2. Animals and Experimental Plan

The detailed experimental design was previously describe in Roda et al. [36]. Briefly, 34 two-month-old wild type female BALB/c mice were obtained from Charles River Italia (Calco, Italy) and acclimatized for at least 3 weeks before the experiments.

All experiments were achieved in agreement with the European Council Directive 2010/63/EU and the Ethics Committee of Pavia University guidelines (Ministry of Health, License number 364/2018-PR). Therefore, all mice have been treated humanely, with due consideration for the reduction of pain and distress.

For execution of experiments and subsequent analyses, researchers were blinded to the designed group.

Sixteen (4T1 M. U-care mice) out of thirty-four mice were supplemented until sacrifice with a medicinal mushroom blend, namely, Micotherapy U-care (provided by A.V.D. Reform s.r.l., Noceto, Parma, Italy) consisting of a mixture of 20% extracts of sporophores and mycelia of five fungal species: *Agaricus blazei*, *Cordyceps sinensis*, *Ganoderma lucidum*, *Grifola frondosa*, and *Lentinula edodes* (see Table 1 and Results section). The mycotherapic blend was solubilized in water, selecting a dose of 4 mg supplement/mice per day to mimic the oral supplementation in humans (about 1.5 g/day). Otherwise, control (*n* = 4) and non-treated (4T1, *n* = 14) mice did not received any diet supplementation.

For the syngeneic tumor-bearing mice (4T1 and 4T1 M. U-care) generation see the work in [36].

The syngeneic tumor-bearing mice (4T1 and 4T1 M. U-care) were generated by injecting 1 × 10^6^ of the 4T1 cells into the nape of the neck of the female Balb/C mice. Control animals were injected with phosphate-buffer saline (PBS). A survival rate of 100% was kept in all experimental groups, throughout the whole experimental time course.

Lung preparation was done by vascular perfusion of fixative [49]. Then, lungs were accurately removed, sectioned and then processed for immunohistochemistry.

### 4.3. Tissue Sampling and Immunohistochemistry

#### 4.3.1. Lung Specimens Preparation

The lung specimens preparation was previously described in detail [36]. Briefly, the top and the bottom regions of the right lungs of mice from each experimental group were dissected. Tissue samples were obtained according to a stratified random sampling scheme, fixed and processed as previously described [36]. Eight micrometer thick sections were cut in transversal plane and placed on silane-coated slides.

#### 4.3.2. TUNEL Staining

The reaction was performed using the terminal deoxynucleotidyl-transferase (TUNEL) assay (Oncogene Res. Prod., Boston, MA, USA). The lung sections were incubated for 5 min with 20 µg mL^−1^ proteinase-K solution at room temperature, followed by treatment with 3% H_2_O_2_ to quench endogenous peroxidase activity. After incubation with the TUNEL solution (90 min with TdT/biotinylated dNTP and 30 min with HRP-conjugate streptavidin) in a humidified chamber at 37 °C, the reaction was developed using a 0.1% DAB solution. After nuclear counterstaining employing Carazzi’s Hematoxylin, the sections were dehydrated in ethanol, cleared in xylene, and finally mounted in Eukitt (Kindler, Freiburg, Germany).

As a negative control, the TdT incubation was omitted; no staining was observed in these conditions.

#### 4.3.3. Immunohistochemistry: Apoptotic Pathway Assessment

Commercial antibodies were employed on murine lung specimens to investigate the expression of different specific apoptotic markers: (i) Poly (AD-ribose) polymerase 1(PARP1), (ii) p53, (iii) Bcl2 associated X-protein (Bax), (iv) B-cell lymphoma/leukemia protein (Bcl2), and (v) the proliferating cell nuclear antigen (PCNA). Table 2 shows both primary and secondary antibodies as well as respective dilutions used for immunohistochemical experiments.

Immunohistochemical procedures have been conducted exactly as previously described [36].

#### 4.3.4. Immunohistochemical Evaluations

To prevent differences due to small procedural changes, immunohistochemical reactions were performed simultaneously on samples from different experimental groups. The expression of each selected marker was examined in six slides (about 30 sections) per mouse. The shown micrographs display the most representative pulmonary conditions and modifications for each immunohistochemical reaction.

Immunohistochemical labeling extent evaluation was previously described in detail [36].

The optical density (OD), intended as immunohistochemical intensity, was assessed in 30 cells/section per six slides/mouse. OD was related to the immunopositive cell density. In addition, immunopositive cells density count was evaluated, intended as number of immunopositive cells/area in mm^2^.

### 4.4. Statistics

Data were expressed as mean ± standard error of the mean (SEM). Regarding the TME, the statistical differences among the three experimental groups were calculated by using one-way ANOVA followed by Bonferroni’s post hoc test. Otherwise, for metastases, the statistical differences between 4T1 and 4T1 M. U-care mice were evaluated by using unpaired Student’s *t*-test.

The differences were considered statistically significant for *p* < 0.05 (*), *p* < 0.01 (**), and *p* < 0.001 (***). Statistical analyses were performed by using GraphPad Prism 7.0 (GraphPad Software Inc., La Jolla, CA, USA).

## 5. Conclusions

Overall, these results support the use of oral supplementation with Micotherapy U-care blend as a new effective strategy to be used in the field of integrative oncology to decrease adverse side effects caused by conventional cancer therapies. All obtained results corroborated the Micotherapy U-care protective role in metastases and TME, in which an immunomodulatory anti-inflammatory systemic action together with a direct, selective anticancer mechanism exerted a positive pleiotropic effect. The Micotherapy U-care preventive and protective effect could affect the TME signaling and, at the same time, target the multifaceted apoptotic pathway. Once again, the present investigation highlights the importance of translational research in the development of clinically relevant therapeutic strategies. In particular, the growing use of translational research “from bench to bedside” in cancer medicine, could allow to overcome challenges which everlastingly hinder medicinal advancements, yielding significant advances in cancer therapeutics and also improvements in the ability to predict clinical course of patient’s disease based on individual tumor characteristics. In this view, medicinal mushrooms extracts, being natural sources of novel drugs, could be used as effective adjuvant therapy in the critical management of TNBC.

## Figures and Tables

**Table 1:** Details on Micotherapy U-care supplement composition.

Medicinal Mushroom	Fungal Part Used in Micotherapy U-Care	% Contained in Micotherapy U-Care	ID Code
*Agaricus blazei*	Fruiting body	20%	7700
*Ophiocordyceps sinensin*	Fruiting body and mycelium	20%	Cm2
*Ganoderma lucidum*	Fruiting body	20%	Gač
*Grifola frondosa*	Fruiting body	20%	Gf3
*Lentinula edodes*	Fruiting body	20%	Le.ed.1

**Table 2:** Primary/secondary antibodies and respective dilution used for immunohistochemical experimental procedures.

	Antigen	Immunogen	Manufacturer, Species, Mono-Polyclonal, Cat./Lot. No., RRID	Dilution
Primary Antibodies	Anti-poly (ADP-ribose) polymerase (46D11)	Purified antibody raised against the residues surrounding Gly623 of human PARP-1	Cell Signaling Technology (Danvers, MA, USA), Rabbit monoclonal IgG, Cat# 9532, RRID:AB_659884	1:100
	Anti-p53 (Ab-5)	Purified antibody raised against the ~53 kDa wild type p53 protein of mouse origin	Sigma-Aldrich (St. Louis, MO, USA), Mouse monoclonal IgG2a, Cat# OP33-100UG, RRID:AB_564977	1:100
	Anti-Bcl-2-associated X protein (P-19)	Purified antibody Raised against a peptide mapping at the amino terminus of Bax of mouse origin	Santa Cruz Biotechnology (Santa Cruz, CA, USA), Rabbit polyclonal IgG, Cat# sc-526, RRID:AB_2064668	1:100
	Anti-B-Cell Leukemia/Lymphoma 2 protein (N-19)	Purified antibody raised against a peptide mapping at the N-terminus of Bcl-2 of human origin	Santa Cruz Biotechnology (Santa Cruz, CA, USA), Rabbit polyclonal IgG, Cat# sc-492, RRID:AB_2064290	1:100
	Anti-Proliferating Cell Nuclear Antigen (Ab-1)	Purified antibody raised against the ~37 kDa PCNA protein of mouse origin	Sigma-Aldrich (St. Louis, MO, USA), Mouse monoclonal IgG2a, Cat# NA03-200UG, RRID:AB_213111	2:1000
Secondary Antibodies	Biotinylated horse anti-mouse IgG	Gamma immunoglobulin	Vector Laboratories (Burlingame, CA, USA), Horse, Cat# PK-6102, RRID:AB_2336821	1:200
	Biotinylated goat anti-rabbit IgG	Gamma immunoglobulin	Vector Laboratories (Burlingame, CA, USA), Goat, lot# PK-6101, RRID: AB_2336820	1:200
